# The functional and structural characterization of a novel oncogene GIG47 involved in the breast tumorigenesis

**DOI:** 10.1186/1471-2407-12-274

**Published:** 2012-07-02

**Authors:** Kyou-Hoon Han, Si-Hyung Lee, Seon-Ah Ha, Hyun Kee Kim, CheWook Lee, Do-Hyoung Kim, Kee Hwan Gong, JinAh Yoo, Sanghee Kim, Jin Woo Kim

**Affiliations:** 1 Division of Biosystems Research, Korea Research Institute of Bioscience and Biotechnology, 125 Gwahak-ro, Yuseong-gu, Daejeon, 305-806, South Korea; 2 Molecular Genetic Laboratory, College of Medicine, The Catholic University of Korea, Seoul, 137-040, South Korea; 3 Department of Obstetrics and Gynecology, College of Medicine, The Catholic University of Korea, Seoul, 137-040, South Korea

**Keywords:** Breast cancer, Oncogene, GIG47, Three-dimensional structure, Anti-cancer agents

## Abstract

**Background:**

A candidate oncogene GIG47, previously known as a neudesin with a neurotrophic activity, was identified by applying the differential expression analysis method.

**Methods:**

As a first step to understand the molecular role of GIG47, we analyzed the expression profile of GIG47 in multiple human cancers including the breast cancer and characterized its function related to human carcinogenesis. Based on this oncogenic role of GIG47, we then embarked on determining the high-resolution structure of GIG47. We have applied multidimensional heteronuclear NMR methods to GIG47.

**Results:**

GIG47 was over-expressed in primary breast tumors as well as other human tumors including carcinomas of the uterine cervix, malignant lymphoma, colon, lung, skin, and leukemia. To establish its role in the pathogenesis of breast cancer in humans, we generated stable transfectants of MCF7 cells. The ectopic expression of GIG47 in MCF7 cells promoted the invasiveness in the presence of 50% serum. In addition, it also resulted in the increased tumorigenicity in *in vivo* tumor formation assay. The tumorigenesis mechanism involving GIG47 might be mediated by the activation of MAPK and PI3K pathways. These results indicate that GIG47 plays a role in the breast tumorigenesis, thus representing a novel target for the treatment of breast cancer. To facilitate the development of GIG47-targeted therapeutics, we determined the structural configuration of GIG47. The high-resolution structure of GIG47 was obtained by combination of NMR and homology modeling. The overall structure of GIG47 has four *α*-helices and 6 *β*-strands, arranged in a *β*1-*α*1-*β*2-*β*3-*α*2-*β*4-*α*3-*α*4-*β*5-*β*6 topology. There is a potential heme/steroid binding pocket formed between two helices *α*2 and *α*3.

**Conclusion:**

The determined three-dimensional structure of GIG47 may facilitate the development of potential anti-cancer agents.

## Background

Breast cancer is nowadays the most frequent malignant tumor in female, and morbidity and mortality continue to increase [1]. Despite advances in early detection and the understanding of the molecular bases of breast cancer biology, about 30% of patients with early-stage breast cancer have recurrent disease. Furthermore, the course of the disease and response to treatment vary greatly [2]. In view of the heterogeneity of breast carcinomas, the need for reliable diagnostic or therapeutic markers is obvious in terms of both their biological profile and their clinical outcome.

The identification of molecular alterations in cancerous and pre-cancerous cells has provided insight into the role of oncogenes and tumor suppressor genes in tumor initiation and progression [3]. Oncogenes are derived from highly conserved protooncogenes that are altered by chromosomal point mutations, gene amplifications, or gene arrangements [4]. The signal transduction pathways subverted by oncoproteins govern fundamental cell functions, including proliferation, cell cycle regulation, and apoptosis [5]. Thus, the structural and functional characterization of oncoproteins underlying cancer will aid in developing the diagnostic and therapeutic avenues.

We measured gene expression levels in a series of breast normal and tumor primary tissues to discover genes involved in tumorigenesis of human breast tissue, and isolated the new human breast cancer-related gene, GIG47 (GenBank accession number AY762102). GIG47 corresponds to Homo sapiens neuron derived neurotrophic factor (NENF) (GenBank accession number NM_013349), which encodes a neurotrophic factor involved in neuron differentiation and development in mice [6,7]. Mouse neudesin is a secreted protein with neurotrophic activity in neurons and undifferentiated neural cells [6,7]. It exhibited a high similarity (∼90% identity) to human and rat neudesins. The neurotrophic activity of neudesin was mediated through the activation of mitogen-activated protein (MAP) [8] and phosphatidylinositol 3-kinase (PI-3 K) [9] pathways.

There are also previous reports on the functional aspect of GIG47 related to the tumorigeneis. According to these groups, GIG47 mRNA was up-regulated in immortalized cells [10]. In agreement with this, the breast cancer proteomics approach demonstrated that GIG47 protein was abundant in estrogen receptor (ER)+/progesterone receptor (PR) + breast cancers [11]. In spite of this previous research on GIG47 related to human cancer, its molecular function remains to be discovered.

As a first step to understand the molecular role of GIG47, we analyzed the expression profile of GIG47 in multiple human cancers including the breast cancer. This result revealed that GIG47 is highly up-regulated in most of human cancers. In addition, the ectopic expression of GIG47 promoted the invasiveness and tumorigenesis of breast cancer cells. Based on this oncogenic role of GIG47, we then embarked on determining the high-resolution structure of GIG47. Bioinformatics predictions and preliminary circular dichroism spectropolarimetry data indicated that GIG47 might belong to the family of intrinsically unfolded proteins (IUPs) with ~50% of its amino acid residues not participating in forming secondary and/or tertiary structure. As IUPs do not produce suitable single crystals for structural study by x-ray crystallography, we have applied multidimensional heteronuclear NMR methods to GIG47 that are currently best suited for characterizing structural features of IUPs [12-14].

The purpose of our study was to identify an unique gene that shows cancer-associated expression, to characterize its function related to human carcinogenesis, and to determine its three-dimensional structure.

## Methods

### Tissues and cell lines

For differential display of mRNA, normal and neoplastic breast tissue specimens were obtained from the surgical services of Gangnam St. Mary’s Hospital, The Catholic University of Korea (Seoul, Korea). Patient written consents were obtained from each individual and the use of tissue samples was approved by the ethics committee of our institution (Catholic ethics committee). Mammalian cell lines described below were all obtained from the American Type Culture Collection (Manassas, VA): MCF-7 and MDA-MB-231 cells are human breast adenocarcinoma. MCF-7 cell line was cultured in Eagle’s Minimum Essential Medium supplemented with 10% FBS and bovine insulin (0.01 mg/ml). MDA-MB-231 cell line was cultured in Leibovitz’s L-15 medium supplemented with 10% FBS.

### Differential display reverse transcription-PCR (DDRT-PCR)

Total RNA was extracted from tissues with an RNA extraction kit (RNeasy total RNA kit, Qiagen Inc., Valencia, CA), and 0.2 μg of total RNA were used to generate cDNA in a reverse transcription reaction (RNAimage kit, GenHunter, Nashville, TN). With the use of the differential display kit (RNAimage kit), we performed PCR with oligo-dT primers and arbitrary sequences, each 13 bases in length according to the manufacturer’s recommendations. After cDNA of mRNAs was generated, the PCR products were separated by electrophoresis on a 6% denaturing polyacrylamide gel. The cDNA was then reamplified without [α-^35^ S]dATP and with 20 μM deoxynucleotide triphosphates instead of 2 μM deoxynucleotide triphosphates. From the films, a 206-bp cDNA (referred to as HP73) was identified by the use of 5’-arbitrary primer H-AP21 (5’-AAGCTTTCTCTGG-30) and 3’ H-T11C anchored primer (5’-AAGCTTTTTTTTTTTC-3’; GenHunter). HP73 was then subcloned into the pGEM-T easy vector with the use of the TA cloning system and sequenced to an automatic sequencing analysis [15,16].

### cDNA library screening

To isolate the full-length cDNA clone that contained the partial HP73 sequence, a bacteriophage λgt11 human lung embryonic fibroblast cDNA library was screened by plaque hybridization with ^32^P-labeled HP73 partial cDNA probe.

### Construction of expression vector, DNA transfection, and western blotting

To generate an eukaryotic expression construct of GIG47, we amplified the coding region of GIG47 by PCR, which was then subcloned into pcDNA3.1 plasmid (pcDNA3.1 Directional TOPO expression kit, Invitrogen) according to the manufacturer’s recommendations. To express GIG47 in MCF7 cells, we seeded 3x10^5^ cells per 60-mm tissue culture dish (Costar, Cambridge, MA). The next day, the cells were transfected with 10 μl of Lipofectamine 2000 reagent (Invitrogen, CA) and 5 μg of GIG47/pcDNA3.1 and pcDNA3.1 vector alone. After a 10-h incubation, the cells were cultured in media supplemented with 10% FBS.

For Western blot analysis, cells were lysed in a lysis buffer [20 mM Tris (pH 7.4), 1% NP40, 5 mM EDTA, 10% glycerol, 0.1% SDS, and 150 mM NaCl] containing protease inhibitor mixture (Sigma, St. Louis, MO). The cell lysates containing 20 μg of total protein were loaded on 10% SDS-polyacrylamide gels and separated by electrophoresis. Protein samples were then transferred to nitrocellulose membranes. The blots were incubated with polyclonal anti-V5 antibody (Invitrogen) and developed with the enhanced chemiluminescence detection kit (Amersham Pharmacia Biotech., Uppsala, Sweden).

### Northern blot analyses

Total RNA was extracted from frozen human tissues with TRIzol reagent (Invitrogen). Northern blot analysis were carried out in which 20 μg of denatured total RNA were electrophoresed on a 1.0% formaldehyde agarose gel and transferred to a nylon membrane (Roche Diagnostics GmbH, Mannheim, Germany). The mRNA expression of GIG47 was also assessed in normal human tissues and a variety of human cancer cell lines with the use of membrane commercially available from Clontech (Palo Alto, CA), which was processed as recommended by the supplier. Human β-actin cDNA control probe provided by Clontech was used as a loading control. All blots were hybridized with the randomly primed [^32^P]-labeled GIG47 partial cDNA probe (the HP73 fragment).

### Quantitative RT-PCR analysis

Total RNA was extracted from different cell lines and normal breast tissues using the RNAeasy minikit (Qiagen). The quality of the RNA was determined on agarose gel electrophoresis. After the spectrometric analysis, equal amounts of RNA were used for cDNA synthesis. After DNase treatment, oligo dT primers were used for first-strand cDNA synthesis. All procedures were performed according to the manufacturer’s instruction (Invitrogen). Quantitative PCR was performed on the CFX96 Real-time PCR Detection System using iQ SyberGreen supermix according to instructions and analyzed by software (all Bio-Rad Laboratories, CA). The primers were designed by BEACON DESIGNER (v7.5; Premier Biosoft International). Sequences were as follows: GIG47 forward: 5’-TGGCAGTGAAGGGAGTGGTGTTTG-3’, reverse: 5’- CCGTAGTGTCATGGGTGAGGTCTG-3’; β-actin forward: 5’-CTCTTCCAGCCTTCCTTCCT-3’, reverse: 5’-AGCACTGTGTTGGCGTACAG-3’.

### Invasion assay

Invasion assays were done using the Cell Invasion Assay kit (Chemicon International, Inc., CA). Briefly, cells (5,000/ml) were maintained in serum-free medium with or without 50% FBS as a chemoattractant at 37 °C for 24 hours. Invaded cells were subsequently detached, lysed, and detected by CyQuant GR dye using fluorescence plate reader Mithras LB 940 (Berthold Technologies, Germany).

### Tumorigenicity assay

5-week-old athymic nude mice (4–8 per group) were injected s.c. with 10^6^ MCF7-GIG47 and MCF7-pcDNA3.1 cells into axillary mammary fat pads. Tumors were measured weekly and tumor areas were calculated as 1/4 x length x width x π. The data is reported as mean tumor area per group.

### Protein purification

In order to express and purify GIG47, we have subcloned the synthesized GIG47 gene into a V3 expression vector, using Nhe I (GCTAGC) and Xho I (CTCGAG). Also, to enhance the solubility for facile purification of GIG47 and for sample preparation for NMR measurements we added his-tags to both N- and C- termini with the sequences of MHHHHHHSSGLVPRGSGMKETAAAKFERQHMDSPDLGTDDDDKAMASGG and GGGLEHHHHHH, respectively. Transformed *Escherchia coli* BL21(DE3) cells were grown at 37 °C to an OD_600_ of 0.6 and the culture was induced with 0.5 mM isopropyl thio-β-D-thiogalactopyranoside (IPTG). Then, the cells were further cultivated at 20 °C for 16 hours. The harvested cell suspension was sonicated in 20 mM Tris–HCl (pH 8), 0.5 M NaCl, 1 mM PMSF, 20 mM imidazole and centrifuged for 30 minutes at 30,000 × g. Both unlabeled and ^13^ C/^15^ N-labeled GIG47 were purified, using Ni-Sepharose column, Q-Sepharose column, SP-Sepharose column, and Hiprep 26/60 Sephacryl S-200 FPLC column (GE Healthcare). The molecular weight of the purified protein was confirmed by MALDI-TOF mass spectrometry.

### NMR spectroscopy

NMR spectra were acquired using a Varian Unity INOVA 600 and Bruker Avance II 900 spectrometers equipped with cryogenic probes. For backbone assignment of the GIG47, ^15^ N-^1^ H HSQC, gnoesyNhsqc, gtocsyNhsqc, HNCACB, CBCACONNH, HNCO, HNCA, CCC-TOCSY, and HNCACO spectra were obtained at 30 °C in 20 mM sodium acetate-d_3_ (pH 6.5), 0.01 mM EDTA, 0.1 mM PMSF, 1 mM DTT, 0.1 mM benzamidine, 0.02% (w/v) NaN_3_, 90% H_2_O/10% ^2^H_2_O. The C_α_ CSI values were obtained based on the method of Wishart and Sykes [17-19]. All data were processed and analyzed using a Varian Vnmr software and an nmrPipe/nmrDraw software.

### CD spectropolarimetry

A CD spectrum was obtained with a 20 μM protein sample on a JASCO J-720 spectropolarimeter using a 1 mm cell in 20 mM sodium acetate (pH 6.5), 0.1 mM PMSF, 0.01 mM EDTA, 1 mM DTT, 0.1 mM benzamidine, 0.02% (w/v) NaN_3_ at 20 °C.

### Homology modeling

The high-resolution three-dimensional structure of GIG47 was determined by a homology modeling procedure MODELLER using 1TOG as a template according to a published protocol [20]. 1TOG is a hypothetical protein At2g24940.1 found *Arabidopsis thaliana* (BMRB id: bmrb 6138) whose function is unknown [21]. Since the sequence homology between the two proteins is ~ 49% (outside the twilight zone) calculating the structure of GIG47 by homology modeling is a reasonable strategy.

## Results

### Cloning of GIG47 over-expressed in the breast cancer and GIG47 gene expressions in human cancers

**Figure 1:**
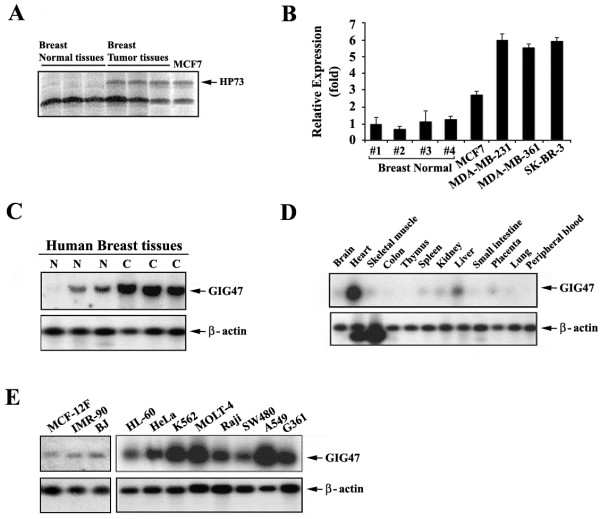
**Identification of GIG47 and its expression patterns in human breast cancers, multiple human tissues and cancer cell lines.** (**A**) Comparative gene expression profile examined by DDRT-PCR using total RNA isolated from normal breast tissue, breast cancer tissue, and MCF-7 breast cancer cell line. Differential display was carried out 5' arbitrary primer H-AP21 (5’-AAGCTTTCTCTGG-3’) and 3’ H-T11C anchored primer (5’-AAGCTTTTTTTTTTTC-3’; GenHunter). The PCR products were resolved by electrophoresis. HP73 is the name of the partial GIG47 gene product. The arrow identifies the location relative to other PCR products. (**B**) The expression level of GIG47 was examined by the quantitative PCR analysis in various breast cancer cell lines compared to the normal breast tissues. (**C**) Total RNAs were isolated from normal breast tissues and their corresponding primary breast cancer tissues. Blot was hybridized with the randomly primed [^32^P]-labeled GIG47 partial cDNA probe (the HP73 fragment). Human β-actin cDNA was used as a control probe (lower panel). Northern blotting was performed to determine the expression of GIG47 in different human tissues. Normal 12 lane multiple tissue northern blot (**D**) or human cancer cell line multiple northern-blot (E) purchased from Clontech were probed with a radioactively labeled HP73 partial cDNA (upper panel) or human β-actin cDNA control probe provided by Clontech (lower panel). (**E**) The normal cell lines included here were BJ, IMR-90, and MCF12F which are a human fibroblast from normal foreskin, a human lung cell fibroblast, and an epithelial cell line from normal mammary gland, respectively.

To discover genes critical for the breast tumorigenesis, we analyzed the differential gene expression profile by applying a differential display technique. Total RNAs were isolated from the normal breast tissues, the breast cancer tissue, and the breast cancer cell line (MCF-7). Partial cDNA synthesized from them was resolved on acrylamide gel and excised from the gel based on their cancer-associated expressions. We then identified the 206-bp partial cDNA fragment named HP73 that was over-expressed in breast cancer tissues and MCF-7 cells but not in normal breast tissue (Figure 1A). Using the partial cDNA HP73 as a probe, we next screened human lung embryonic fibroblast cDNA library to isolate the full-length cDNA. One clone with a size of 703-bp named GIG47 (GenBank accession number AY762102) was isolated. Sequence analysis showed that this cDNA encodes NENF gene (GenBank accession number NM_013349) with a molecular weight of ~ 19 kDa.

To characterize its expression profile related to the breast cancer, we performed the quantitative PCR analysis in breast normal tissues and several breast cancer cell lines. As consistent with the result in Figure 1A, its expression was highly elevated in all breast cancer cell lines examined here compared to normal breast tissues (Figure 1B).

To assess whether our findings obtained from analyzing the breast cancer cells have physiological and clinical relevance, we examined GIG47 expression in tissue samples derived from primary breast cancers. The expression of GIG47 mRNA in breast tumor tissues was analyzed by Northern blotting on a breast normal and tumor tissue blot. We then observed that GIG47 mRNA level is highly up-regulated in the breast tumors compared to the normal breast tissue (Figure 1C). Therefore, our result suggests that GIG47 over-expressed in human breast cancer might play an important role in the breast tumorigenesis.

Northern hybridization was performed on a human normal multiple tissue mRNA blot and a human cancer cell line mRNA blot to analyze the GIG47 expressions in various normal tissues and cancer cell lines. The GIG47 mRNA (~ 1.0 Kb) was weakly expressed in many normal tissues, including the brain, skeletal muscle, colon, thymus, spleen, kidney, liver, small intestine, placenta, lung and leukocyte except normal heart in which GIG47 transcript was strongly expressed (Figure 1D). In contrast, its level was detected at a very high level in several cancer cell lines such as the promyelocytic leukemia cell line HL-60, cervical cancer cell line HeLa, a chronic myelogenous leukemia cell line K562, lymphoblastic leukemia cell line MOLT-4, Burkitt’s lymphoma cell line Raji, colon cancer cell line SW480, lung cancer cell line A549, and melanoma cell line G361 (Figure 1E). Thus, Northern blot analyses show that GIG47 mRNA exists abundantly in many cancer cell lines compared with its little or no expression in most normal tissues. This suggests that the functional role of GIG47 is required for various cancer cell types derived from different organs. We also added the expression profile of normal cell lines (Figure 1E). The normal cell lines included here are BJ, IMR-90, and MCF12F which are a human fibroblast from normal foreskin, a human lung cell fibroblast, and an epithelial cell line from normal mammary gland, respectively. As shown in the Figure 1E, the expression levels of GIG47 in human normal cell lines were relatively low.

### Ectopic expression of GIG47 results in the increased tumorigenecity

**Figure 2:**
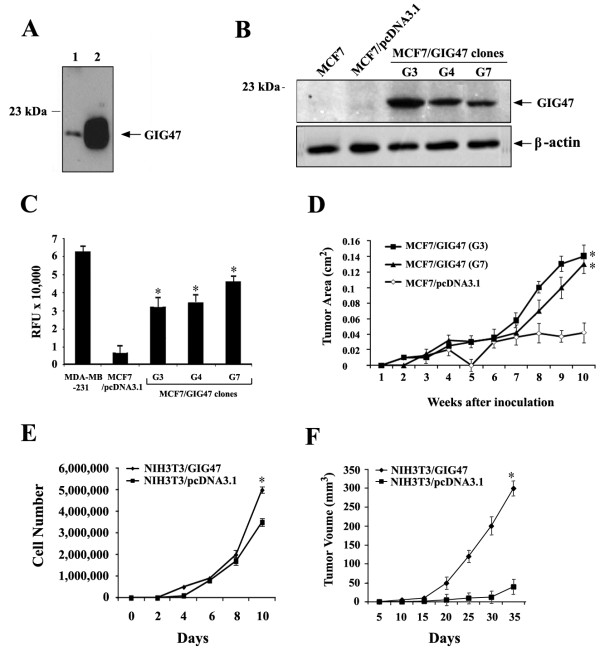
**Increased invasiveness and tumorigenicity of MCF7 stable cell lines over-expressing GIG47.** (**A**) MCF7 cells were transiently transfected with GIG47/pcDNA3.1 construct, and the cell pellet and culture media were separated by centrifugation. The cell pellet was lysed by a lysis buffer while culture media was concentrated. The GIG47 protein expression was analyzed by Western blot analysis with anti-V5 antibodies. Lane 1 and 2 are the cell lysate and concentrated culture media, respectively. (**B**) Western blot analysis of GIG47 protein levels in established MCF7 stable cell lines. (**C**) Effect of GIG47 over-expression in *in vitro* invasion of MCF7 cells. Invasion analysis of three clones was performed in serum-rich medium (50% FBS). Experiments were repeated at least three times. MDA-MB-231 cells were used as a positive control. (**D**) Increased tumorigenicity of GIG47 over-expressing MCF7 cells. MCF7-GIG47 clones (G3 and G7) and MCF7-pcDNA3.1 cells were inoculated into mammary fat pads of athymic nude mice. The tumor growth was measured every week after the inoculation. (**E**) The cell growth rate of NIH/3 T3 overexpressing GIG47 was significantly increased compared to control cells. (**F**) GIG47-overexpressing NIH/3 T3 clones exhibited the increased tumor growth rate compared with NIH/3 T3 cells vector control.

As previously reported [6], GIG47 was a secreted protein mostly expressed in the extracellular supernatant of cells (Figure 2A). To investigate the role of GIG47 in breast cancer, we stably transfected MCF7 cells with pcDNA3.1 and pcDNA3.1-GIG47 constructs, and selected three clones with high steady-state levels of GIG47 (G3, G4, and G7). Western blot analysis showed the bands of expected molecular weight in the cell extracts from MCF7-GIG47 clones but not from vector controls and parental cell types (Figure 2B).

As shown in Figure 2C, MCF7-GIG47 cells in the presence of chemoattractant (50% FBS) showed significantly increased invasiveness almost reaching the level of MDA-MB-231 cells used as a positive control whereas the invasiveness of MCF7 cells stably transfected with the vector alone remained low. All three MCF7-GIG47 clones showed the increased invasiveness compared to MCF7 control cells (Figure 2C).

To test the effect of GIG47 over-expression on tumorigenicity, two GIG47-over-expressing MCF7 clones (G3 and G7) as well as MCF7 cells stably transfected with pcDNA3.1 vector were bilaterally injected into the mammary fat pads of athymic nude mice. Both GIG47-overexpressing MCF7 clones showed increased tumor growth rate compared with MCF7 vector control (Figure 2D). Especially at week 8, tumors formed by MCF7-GIG47 cells were clearly larger than in the control group. Therefore, our result indicates that GIG47 over-expressed in multiple human cancers indeed serves to mediate the tumorigenesis in breast cancer.

We also perform the experiments to suggest the important function of GIG47 related to the tumorigenesis. To do this, we induced the over-expression of GIG47 in NIH/3 T3, a normal murine fibroblast cell line. We then tested its tumorigenecity with the proliferation and *in vivo* tumorigenecity assays. As shown in Figure 2E, the results show that the cell growth rate of NIH/3 T3 is significantly increased compared to control cells. In addition, the clonal population of NIH/3 T3 cells over-expressing GIG47 was bilaterally injected into the mammary fat pads of athymic nude mice together with NIH3T3 cells carrying the empty vector. We then assessed its tumor growth rate. The results showed that GIG47-overexpressing NIH/3 T3 clones exhibit the increased tumor growth rate compared with NIH/3 T3 cells vector control (Figure 2F). Especially at day 30, tumors formed by NIH3T3-GIG47 cells were clearly larger than in the control group. Therefore, these two additional experiments strongly support our conclusion that GIG47 over-expressed in multiple human cancers mediates the tumorigenesis.

### The activation of MAP and PI-3 kinase pathways and the effect of GIG47 knockdown on the cell invasion

**Figure 3:**
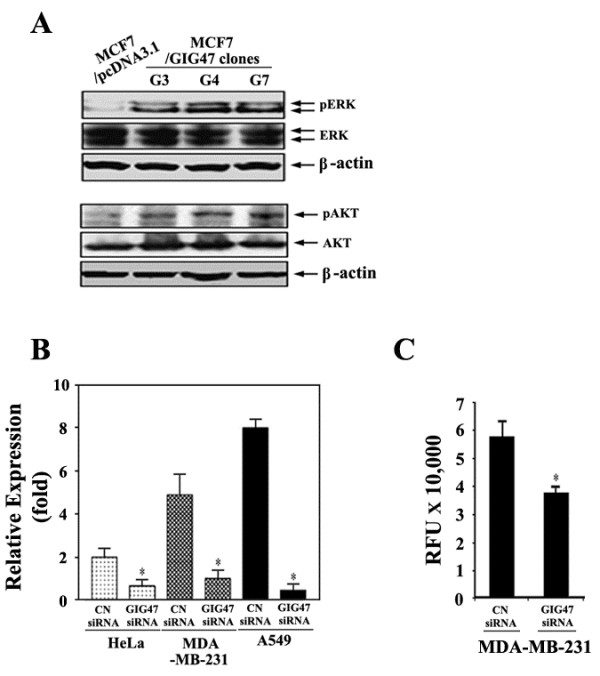
**The activation of MAP and PI-3 kinase pathways and the effect of GIG47 knockdown on the cell invasion.** (**A**) Cell lysates were prepared and quantified for protein content. A total of 100 μg protein was resolved on 10% SDS-PAGE followed by immunoblot analysis with specific antibodies against total or phosphorylated forms of ERK and AKT. Immunoblot for β-actin was done as a loading control. (**B**) GIG47 mRNA levels were measured by real-time PCR. (**C**) Invasion analysis of MDA-MB-231 cells transfected with GIG47 siRNA and control siRNA was performed in serum-rich medium (50% FBS). Experiments were repeated at least three times.

To gain insight into the mechanism underlying the proliferative and/or tumorigenic effect of human GIG47, we examined the changes in signal transduction pathways. Previous studies demonstrated that MAPK and PI3K pathways are involved in mediating the function of a neudesin, a mouse homologue of GIG47. A neudesin is abundantly expressed in the developing brain and spinal cord in mouse embryos. In addition, this protein exhibits the significant neurotrophic activity in primary cultured mouse neurons. Neudesin activated the mitogen-activated protein (MAP) and phosphatidylinositol-3 (PI-3) kinase pathways. This suggests that the neurotrophic activity exerted by the neudesin is mediated *via* the activation of the MAP and PI-3 kinases. Based on this, we, therefore, analyzed the phosphorylation status of ERK and AKTs. Total cellular proteins were extracted from cells over-expressing GIG47 and ones carrying the empty vector, and lysates were immunoblotted with specific phosphorylated antibodies (Figure 3A). We then found that an increased phosphorylation of ERK and AKT was observed in all three clones over-expressing GIG47 while not in control cells. In contrast, GIG47 had no effect on total ERK and AKT protein expression levels. This data suggest that the tumorigenesis mechanism involving GIG47 might be mediated by the activation of MAPK and PI3K pathways.

To further investigate the role of GIG47 in tumorigenecity, we performed the GIG47 knock-down experiments. The baseline expression of GIG47 was determined in a panel of human cancer cell lines as shown in Figure 1E. The results showed that GIG47 was abundantly expressed in most of human cancer cell lines. We then examined the relative mRNA levels of GIG47 in HeLa, MDA-MB-231, and A549 cells by real-time PCR analysis. All three cell lines expressed high levels of GIG47 in mRNA level (Figure 3B).

To determine whether GIG47 could be an effective therapeutic target for the cancer, the effect of GIG47 on cell tumorigenecity was examined. The efficacy of GIG47 siRNA for knockdown of GIG47 mRNA was confirmed by real-time PCR. We observed that GIG47 mRNA levels were significantly lowered in GIG47 siRNA-transfected cells (Figure 3B). The cell invasion was determined in MDA-MB-231 cells transfected with GIG47 siRNA. We then found that the down-regulation of GIG47 expression inhibited the cell invasion in MDA-MB-231 cells (Figure 3C). Therefore, all the data support our conclusion that GIG47 over-expressed in multiple human cancers mediates the tumorigenesis.

### An overall structural analysis of GIG47 by CD and NMR techniques

**Figure 4:**
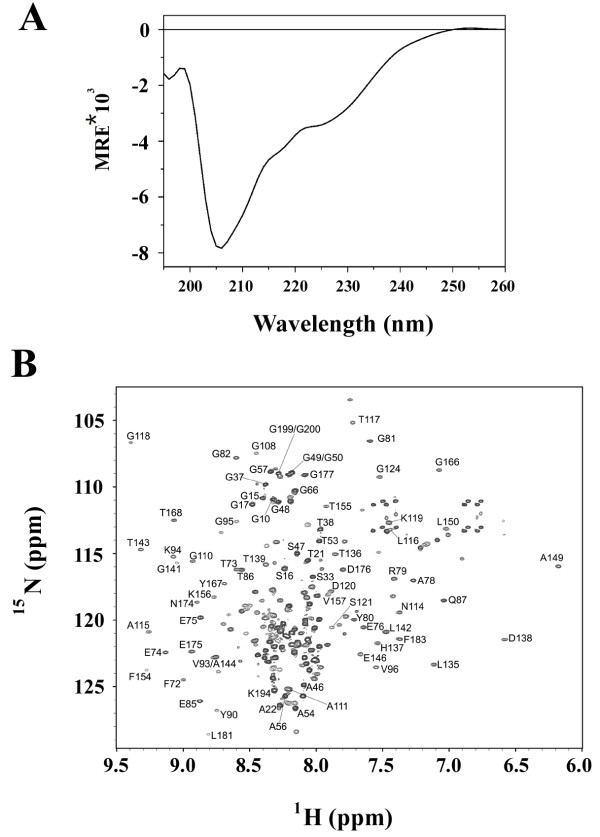
**Overall structural analysis of GIG47 by CD and NMR techniques.** (**A**) A CD spectrum of GIG47 (20 μM) obtained under 20 mM sodium acetate (pH6.5), 0.1 mM PMSF, 0.01 mM EDTA, 1 mM DTT, 0.1 mM benzamidine, 0.02% (w/v) NaN_3_ at 20°C. Low ellipticity at 220 nm indicates that the protein is mostly unstructured. (**B**) An ^15^ N-^1^ H HSQC spectrum of GIG47 (500 μM) obtained under 20 mM sodium acetate-d_3_ (pH 6.5), 0.1 mM PMSF, 0.01 mM EDTA, 1 mM DTT, 0.1 mM benzamidine, 0.02% NaN_3_, 90% H_2_O/10% ^2^H_2_O at 30°C with assigned resonances indicated.

The native GIG47 is composed of 148 amino acid residues if we exclude the added solubilizing tags. The structural determination of GIG47 might provide an insight on the functional aspect as well as the development of cancer therapeutics. The secondary structure prediction on GIG47 shows that this protein contains some secondary structures (~23% helix and ~16% β-conformation by GOR4 predictor). The CD spectrum of GIG47 shown in Figure 4A indicates that it is mostly unstructured. When GIG47 is subjected to GLOBPLOT [22], a program for predicting disordered regions in intrinsically unfolded proteins (IUPs), ~ 60 out of 148 residues are predicted to be unstructured. Similar disordered segments are predicted when GIG47 is analyzed by other IUP prediction programs.

In order to characterize the detailed conformational features of GIG47 we produced an ^15^ N/^13^ C double labeled recombinant GIG47 and analyzed it using heteronuclear multi-dimensional NMR techniques. A representative ^15^ N-^1^ H HSQC spectrum of GIG47 with assigned residues is shown in Figure 4B. With the two solubility-enhancing tags added to both termini of GIG47 the actual size of protein studied by NMR is 208 residues long. Thus, resonance overlap prevented a complete resonance assignment, but ~70% of GIG47 resonances could be assigned at a reliable level (Additional file 1: Table S1).

### Determination of a high-resolution structure of GIG47

**Figure 5:**
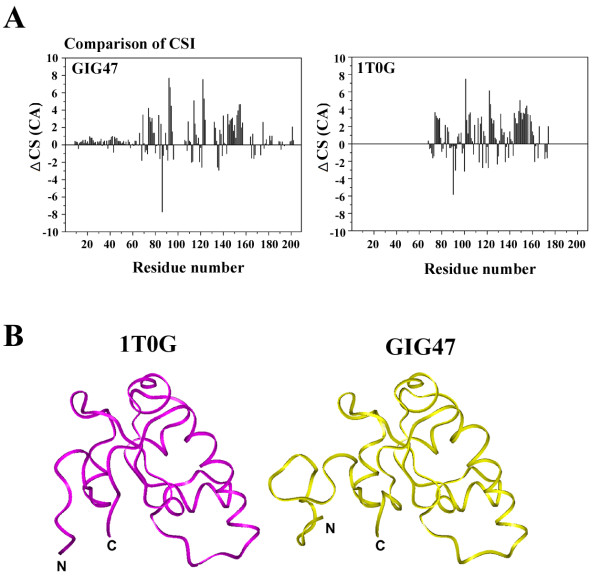
**Secondary structure assignment and homology remodeling.** (**A**) Chemical shift based secondary structure assignment in GIG47 and 1TOG. The overall secondary structure distribution determined by Cα chemical shifts in two proteins is very similar except for the additional solubility tagging regions at the protein termini of GIG47. (**B**) The ribbon diagrams for GIG47 and 1TOG. The structure of GIG47 was obtained by NMR chemical shifts and homology modeling using 1TOG as a template. The two structures are highly superimposable except for the additional solubility tag regions at the protein termini of GIG47.

Secondary structure assignment was possible for the assigned residues as shown in Figure 5A where chemical shift indices for the Cα carbons in GIG47 and its homolog 1TOG [21] are displayed.

Shown in Figure 5B is the structure of GIG47 obtained by a homology modeling calculation [20] using 1TOG as a template. GIG47 consists of four *α*-helices (residues: 25–32, 55–58, 73–78, 95–112, denoted as *α*1 ~ *α*4) and 6 *β*-strands (residues: 23–24, 41–43, 48–50, 70–71, 115–117, 123–124, denoted as *β*1 ~ *β*6), arranged in a *β*1-*α*1-*β*2-*β*3-*α*2-*β*4-*α*3-*α*4-*β*5-*β*6 topology. GIG47 has two *β*-sheets: a short one consists of two *β*-strands arranged in parallel fashion (*β*1 and *β*6 in N- and C-terminal region, respectively) and the other consists of four *β*-strands (*β*4*, β*2, *β*3, and *β*5). Among them, *β*4 and *β*2 form a parallel *β*-sheet and *β*2, *β*3 and *β*5 form an antiparallel *β*-sheet. Three *α*-helices (*α*2, *α*3, and *α*4) lying over the antiparallel *β*-sheet and loop linking *α*2 with *α*3 form a wide pocket, which may play a role of *hydrophobic* binding pocket.

The final structure of GIG47 protein obtained by a combination of homology modeling calculation and NMR chemical shift assisted secondary structure prediction is similar to that of 1T0G. However, there are local structural differences between GIG47 and 1TOG. First, GIG47 has longer helices involving 25E ~ 32 G and 95A ~ 112 K than the corresponding helices (14A ~ 17 L and 84E ~ 98E, respectively) in 1T0G. Second, a short *β*-strand (*β*4) found in GIG47 is absent in 1TOG. Third, a long *β*-strand, V^103^VGRVV^108^, located at the C-terminal region of 1T0G are replaced by two short *β*-strands, 115I ~ 117 G and 123I ~ 124 L in GIG47. Two termini of GIG47 show disordered residues (22 and 23 residues at the N- and the C-terminus, respectively). When combined with a 49-residue solubility-enhancing tag inserted to the N-terminus of GIG47 the actual GIG47 protein studied by NMR contains a 71 disordered residues at its N-terminus.

## Discussion

To discover genes involved in human breast carcinogenesis, we applied DDRT-PCR and identified the candidate human breast cancer-related gene, GIG47 (GenBank accession number AY762102). GIG47 encodes neudesin, a neuron derived neurotrophic factor (NENF) (GenBank accession number NM_013349) in the database.

The neudesin gene has been identified in vertebrates including humans, mice, and zebrafish, but not in invertebrates. This indicates that the neudesin gene is specific to vertebrates. The human neudesin gene is located on chromosome 1 at p33; however, this location has not shown linkage with known inherited diseases [6]. Neudesin is a novel secreted protein with essentially no primary structural homology to any known proteins. Its activity was governed by the activation of the MAP and PI-3 kinase pathways possibly *via* the activation of a Go/Giprotein-coupled receptor. Neudesin is therefore assumed to be a novel neurotrophic factor with a unique structure. Therefore, the study on neudesin may provide new insights into the neuronal development and maintenance [6].

However, study demonstrated that GIG47 mRNA is over-expressed in human immortalized cells [10]. In addition, its protein was also detected as abundant among estrogen receptor (ER)+/progesterone receptor (PR) + breast cancers [11]. Nevertheless, it is unknown how GIG47 contributes to the cellular and biochemical mechanisms of human tumorigenesis. In this study, our result also revealed that GIG47 is over-expressed in various human tumors including carcinomas of the uterine cervix, lymphoma, colon, lung, skin and leukemia, as well as carcinoma of the breast. In a sharp contrast, expression of GIG47 was generally low in diverse human normal tissues. It implies that GIG47 may play an oncogenic role in multiple body organs. In order to address this, we took advantage of ectopic over-expression system and demonstrated that GIG47, indeed, mediates the tumorigenesis of human breast cancer by promoting the invasiveness of breast cancer cells and tumor growth *in vivo*. Therefore, this result strongly supports that GIG47 plays a role in the human tumorigenesis.

Further understanding on GIG47 function and potentially useful information for designing anti-cancer pharmaceuticals may be provided by studying the structural characteristics of GIG47 at an atomic resolution. GIG47 itself shows a poor solubility when it is expressed alone. To obtain a more soluble protein we attached solubility-enhancing tags, which made the total length of the actual protein studied by NMR to consist of 208 residues. Consequently achieving a full resonance assignment was not possible. Nevertheless, as the assigned region of GIG47 has a strong sequence homology of ~50% to a hypothetical protein At2g24940.1 from *Arabidopsis thaliana* (1TOG) with an unknown function (Figure 5), we were able to produce a high-resolution structure of GIG47 by combining NMR chemical shift indices and homology modeling process. Thus our structure determination of GIG47 incidentally made it possible to assign function to a hypothetical protein with an unknown function.

**Figure 6:**
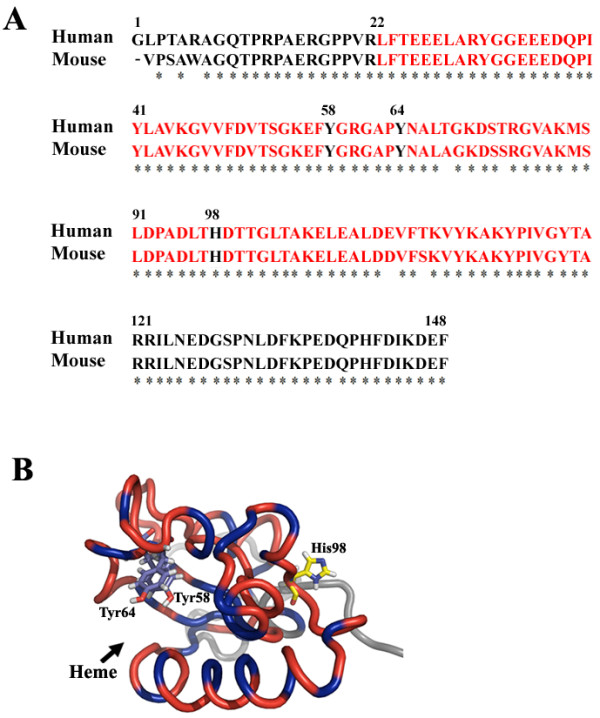
**Comparison of amino acid sequences of GIG47 and NENF and a ribbon diagram of GIG47 showing the potential heme-binding region.** (**A**) Amino acid sequences of GIG47 (human neudesin) and NENF (mouse neudesin) are aligned. The potential heme/steroid binding domain is shown in red. The three residues in bold are the putative heme-binding sites in NENF. (**B**) A ribbon diagram of GIG47 is shown with three potential heme-binding residues (Tyr58, Tyr64 and His98) displayed in a stick representation mode. The heme-binding domain is colored red. The blue regions in the ribbon indicate hydrophobic residues. A potential heme/steroid binding hydrophobic pocket is clearly visible (indicated by an arrow) between two α-helices (α2 and α3).

A structure-based rational drug design strategy including SAR-by-NMR screening was successfully applied in developing potent anti-cancer agents based upon inhibitory activity of mdm2 [23-26]. Since the potential ligand-binding pocket in GIG47 is hydrophobic in nature like that in mdm2, above strategy may also prove to be useful in developing anti-cancer agents using inhibitors against GIG47. A previous work on NENF (mouse neudesin) showed that the protein has a heme/steroid binding region (Leu22 ~ Ala120) [7]. As shown in Figure 6A, the amino acid sequences of NENF and GIG47 are highly homologous (94.6%). In particular the residues in the heme binding region of NENF are exactly the same as those in GIG47 except for 4 residues. Thus we expect that GIG47 (human neudesin) should be able to form a heme/steroid binding region. Three residues (Y58, Y64, H98) in mouse neudesin play an important role in binding with heme. In order to determine potential heme-binding residues in GIG47 we have used the prediction program, HemeBind [27], and found that Phe57, Tyr58, Arg60, Tyr64, Ala66, and Leu67 in GIG47 may interact with heme. The structure of GIG47 we have presented in this work reveal that two residues, Tyr58 and Tyr64, are potential heme-binding sites as shown in Figure 6B, where the side-chains of these two tyrosines are located in the potential heme-binding pocket facing toward the heme. A similar structural pattern is observed in human adrenal inner zone antigen (hIZA), which has a tetrapeptide segment (D_99_VTK_102_) that anchors the heme into the hydrophobic pocket [28]. A similar tetrapeptide segment is found in GIG47, D_50_VTS_53_, thus is expected to carry out a similar role of anchoring a heme group into the hydrophobic pocket of GIG47. In GIG47, a third potential heme-binding residue [7], His98, is located in the opposite side of two Tyr residues and therefore is not likely to contribute to heme binding. The structural analysis described above for GIG47 insinuates that progesterone, a steroid derived from breast tumors, may be the cellular ligand for GIG47.

## Conclusion

In conclusion, GIG47 is over-expressed in various human tumors and plays an oncogenic role especially in the breast cancer. Although further investigation is needed, this result indicates that increased expression of the GIG47 may be associated with human tumorigenesis. Therefore, GIG47 might be a good target for developing diagnostic and therapeutic agents against human cancers when a detailed functional domain is identified using the three-dimensional structure of GIG47 determined in this investigation.

## Competing interests

The authors declare that they have no competing interests.

## Author contributions

Conceived and designed the experiments: KH, JWK. Performed the experiments: KH, SL, SH, HKK, CL, DK, KWG, JY, SK. Analyzed the data: KH, SL, SH, HKK, JWK. Contributed reagents/materials/analysis tools: JY, SK, JWK. Wrote the paper: KH, SH, JWK. All authors read and approved the final manuscript.

## Pre-publication history

The pre-publication history for this paper can be accessed here:

http://www.biomedcentral.com/1471-2407/12/274/prepub

## Supplementary Material

Additional file 1 Table S1. Chemical shift assignments for the 208-residue GIG47 at pH 6.5 and 30 °C. Click here for file
