# Supplementary material for: The functional and structural characterization of a novel oncogene GIG47 involved in the breast tumorigenesis
Source: BMC Cancer. 2012 Jul 2;12:274. doi: 10.1186/1471-2407-12-274 (PMC3411491; doi:10.1186/1471-2407-12-274)
Supplement: Additional file 1 — Table S1. Chemical shift assignments for the 208-residue GIG47 at pH 6.5 and 30 °C. [file 1471-2407-12-274-S1.doc]

**Supplementary Table S1. Chemical shift assignments for the 208-residue GIG47 at pH 6.5 and 30 °C**

Residue ^15^N NH ^13^C^α^ ^13^C^β^ ^13^CO Residue ^15^N NH ^13^C^α^ ^13^C^β^ ^13^CO

Met^1 - - - - -^ Arg^61^ 123.124 8.36 53.912 174.252

His^2 - - - - -^ Pro^62^ 63.289

His^3 - - - - -^ Ala^63^ 124.387 8.326 52.586 177.757

His^4 - - - - -^ Glu^64^ 120.599 8.286 56.508 176.317

His^5 - - - - -^ Arg^65^ 121.984 8.257 55.995 176.217

His^6 - - - - -^ Gly^66^ 110.3 8.16 44.361 170.961

His^7 - - - - -^ Pro^67^ -

Ser^8 - - - - -^ Pro^68^ 60.982 -

Ser^9^ 118.591 8.412 58.668 - 174.779 Val^69^ - - 65.619 -

Gly^10^ 110.98 8.312 45.393 - 173.675 Arg^70^ - - 56.069 -

Leu^11^ 121.981 7.976 55.093 176.932 Leu^71^ - - 53.919 176.987

Val^12^ 122.966 8.011 59.772 174.262 Phe^72^ 124.483 9.001 57.298 176.885

Pro^13^ 63.142 Thr^73^ 116.178 8.603 60.918 175.220

Arg^14^ 122.288 8.384 56.349 176.919 Glu^74^ 122.422 9.137 60.784 179.697

Gly^15^ 110.831 8.402 45.288 174.136 Glu^75^ 119.798 8.873 59.648 178.370

Ser^16^ 116.113 8.241 58.656 175.110 Glu^76^ 120.527 7.642 59.104 179.075

Gly^17^ 111.311 8.482 45.465 174.111 Leu^77^ 118.577 8.271 57.958 177.208

Met^18^ 120.274 8.084 55.661 176.163 Ala^78^ 116.992 7.268 53.537 178.703

Lys^19^ 122.819 8.307 56.575 176.486 Arg^79^ 116.835 7.41 57.223 176.302

Glu^20^ 122.639 8.442 56.821 176.738 Tyr^80^ 119.973 7.716 57.024 174.855

Thr^21^ 115.543 8.067 62.008 174.498 Gly^81^ 106.537 7.592 44.945 176.166

Ala^22^ 126.429 8.274 53.103 177.780 Gly^82^ 107.807 8.604 44.872 173.855

Ala^23^ 122.985 7.999 52.908 177.888 Glu^83^ 121.048 8.123 59.845 176.931

Ala^24^ 122.834 8.091 52.958 177.775 Glu^84^ 117.887 8.46 54.85 177.637

Lys^25^ 119.947 7.977 56.854 176.461 Glu^85^ 126.094 8.88 58.912 176.387

Phe^26^ 120.587 8.013 58.054 175.809 Thr^86^ 116.226 8.563 54.267 175.447

Glu^27^ 122.416 8.166 56.807 176.292 Gln^87^ 118.529 7.038 52.889 173.714

Arg^28^ - - - - Pro^88^

Gln^29^ - - 56.278 175.832 Ile^89^ - - 62.382 173.770

His^30^ 119.654 8.334 55.914 174.518 Tyr^90^ 126.801 8.754 57.325 174.829

Met^31^ 121.848 8.181 55.626 175.469 Leu^91^ 119.193 8.52 53.098 177.633

Asp^32^ 121.66 8.349 54.446 175.702 Ala^92^ 119.664 8.662 59.713 177.027

Ser^33^ 116.745 8.026 56.247 172.797 Val^93^ 122.817 8.777 68.766 176.919

Pro^34^ 63.467 Lys^94^ 115.327 9.079 60.972 175.435

Asp^35^ 120.313 8.25 54.296 176.075 Gly^95^ 112.551 8.601 46.287 176.398

Leu^36^ 123.259 8.078 55.226 177.763 Val^96^ 123.53 7.549 60.313 176.812

Gly^37^ 109.825 8.384 45.458 174.251 Val^97^ - - -

Thr^38^ 113.225 7.97 61.573 174.349 Phe^98^ - - -

Asp^39^ 122.836 8.393 54.551 175.951 Asp^99^ - - -

Asp^40^ - - 54.728 - Val^100^ - - -

Asp^41^ - - 55.044 - Thr^101^ - - -

Asp^42^ - - 55.044 174.297 Ser^102^ - - -

Lys^43^ 121.552 8.062 55.701 177.037 Gly^103^ - - -

Ala^44^ 123.751 8.052 53.201 178.276 Lys^104^ - - -

Met^45^ 118.819 8.05 55.784 - 176.453 Glu^105^ - - -

Ala^46^ 124.879 8.093 52.856 177.905 Phe^106^ - - 58.285 -

Ser^47^ 115.002 8.146 58.605 175.196 Tyr^107^ - - 58.451 175.628

Gly^48^ 111.107 8.287 45.576 174.711 Gly^108^ 107.482 8.451 44.316 172.190

Gly^49^ 109.052 8.209 45.4 174.518 Arg^109^ 120.67 8.644 58.683 178.165

Gly^50^ 108.959 8.183 45.041 173.634 Gly^110^ 115.637 8.933 45.295 179.740

Leu^51^ 122.985 8.023 53.249 175.579 Ala^111^ 125.2 8.203 50.515 -

Pro^52^ 63.441 Pro^112^ 60.817

Thr^53^ 114.058 7.979 61.931 174.378 Tyr^113^ - - 56.066 176.387

Ala^54^ 126.628 8.16 52.601 177.505 Asn^114^ 119.431 7.374 57.698 177.503

Arg^55^ 120.742 8.163 55.928 175.989 Ala^115^ 120.919 9.264 54.782 174.841

Ala^56^ 125.695 8.233 52.777 178.145 Leu^116^ 113.207 7.439 56.179 177.967

Gly^57^ 108.827 8.339 45.327 173.997 Thr^117^ 105.165 7.726 62.194 176.279

Gln^58^ 119.996 8.087 55.602 175.856 Gly^118^ 106.687 9.399 45.821 173.974

Thr^59^ 118.96 8.207 60.062 172.641 Lys^119^ 112.745 7.449 54.53 173.368

Pro^60^ 63.153 Asp^120^ 117.818 7.889 53.484 175.339

**Table S1.** *Continued*

Residue ^15^N NH ^13^C^α^ ^13^C^β^ ^13^CO Residue ^15^N NH ^13^C^α^ ^13^C^β^ ^13^CO

Ser^121^ 120.523 7.88 55.716 174.943 Asp^182^ 118.317 8.073 54.203 175.99

Thr^122^ 118.923 8.506 69.618 174.524 Phe^183^ 121.432 7.37 59.083 174.342

Arg^123^ 120.303 8.703 61.446 176.766 Lys^184^ - - -

Gly^124^ 109.246 7.522 47.654 176.704 Pro^185^ -

Val^125^ - - - Glu^186^ - - -

Ala^126^ - - - Asp^187^ - - -

Lys^127^ - - - Gln^188^ - - -

Met^128^ - - - Pro^189^ 63.377

Ser^129^ - - - His^190^ 117.752 8.33 55.65 174.252

Leu^130^ - - - Phe^191^ 121.886 7.918 57.374 174.726

Asp^131^ - - - Asp^192^ 122.82 8.325 54.117 175.690

Pro^132^ 65.717 Ile^193^ 122.282 8.034 61.358 176.047

Ala^133^ 119.32 8.564 54.181 178.534 Lys^194^ 125.248 8.315 56.353 176.092

Asp^134^ 114.195 8.298 54.319 175.662 Asp^195^ - - -

Leu^135^ 123.315 7.111 52.627 174.427 Glu^196^ - - -

Thr^136^ 115.082 7.855 58.998 171.074 Phe^197^ - - -

His^137^ 121.737 7.539 57.341 175.690 Gly^198^ - - -

Asp^138^ 121.442 6.573 55.117 176.817 Gly^199^ - - 45.375 174.269

Thr^139^ 115.846 8.379 60.929 175.135 Gly^200^ 109.034 8.283 45.375 174.320

Thr^140^ - - 65.493 176.364 Leu^201^ 121.479 8.063 56.997 177.559

Gly^141^ 115.652 9.052 45.255 175.220 Glu^202^ 120.449 8.339 56.97 176.223

Leu^142^ 120.881 7.47 55.049 177.650 His^203^ - - -

Thr^143^ 114.706 9.325 60.827 175.046 His^204^ - - -

Ala^144^ 122.785 8.756 55.743 180.740 His^205^ - - -

Lys^145^ 117.413 8.031 58.814 179.661 His^206^ - - -

Glu^146^ 122.579 7.666 59.187 178.692 His^207^ - - -

Leu^147^ 120.512 8.4 57.929 179.715 His^208^ - - -

Glu^148^ 114.503 8.374 59.483 178.601

Ala^149^ 115.982 6.174 53.767 178.204

Leu^150^ 113.142 7.019 57.338 176.065

Asp^151^ 119.999 8.037 54.666 172.507

Glu^152^ - - 59.946 178.222

Val^153^ 119.431 8.44 65.978 178.936

Phe^154^ 123.755 9.283 62.483 175.232

Thr^155^ 111.486 7.924 66.621 175.850

Lys^156^ 118.293 8.775 58.398 176.710

Val^157^ 118.104 7.915 64.515 175.838

Tyr^158^ - - - -

Lys^159^ - - - -

Ala^160^ - - - -

Lys^161^ - - - -

Tyr^162^ - - - -

Pro^163^ - -

Ile^164^ - - 61.95 -

Val^165^ - - 60.638 175.747

Gly^166^ 108.729 7.074 46.025 169.194

Tyr^167^ 117.205 8.693 56.358 174.988

Thr^168^ 112.497 9.072 60.73 175.860

Ala^169^ - - - -

Arg^170^ - - - -

Arg^171^ - - - -

Ile^172^ - - - -

Leu^173^ - - 53.778 177.610

Asn^174^ 118.724 8.902 52.613 177.559

Glu^175^ 122.381 8.94 59.344 176.630

Asp^176^ 116.213 7.8 53.509 177.057

Gly^177^ 109.075 8.087 45.301 173.346

Ser^178^ 117.943 8.095 56.724 171.54

Pro^179^ -

Asn^180^ - - 52.567 176.919

Leu^181^ 128.555 8.812 56.279 177.406
